# DNA methylation and expression of the *egfr* gene are associated with worker size in monomorphic ants

**DOI:** 10.1038/s41598-022-25675-4

**Published:** 2022-12-08

**Authors:** Thibaut Renard, Cyril Gueydan, Serge Aron

**Affiliations:** 1 Evolutionary Biology and Ecology, Université Libre de Bruxelles, Avenue F.D. Roosevelt, 50, 1050 Brussels, Belgium; 2 Molecular Biology of the Gene, Université Libre de Bruxelles, Rue Prof. Jeener et Brachet, 12, 6041 Gosselies, Belgium

**Keywords:** Epigenetics, Transcription, Development, Animal physiology

## Abstract

The reproductive division of labour is a hallmark of eusocial Hymenoptera. Females are either reproductive queens or non-reproductive workers. In ants, workers often display further task specialisation that is associated with variation in size and/or morphology. Because female polyphenism is typically under environmental control, it is thought epigenetic mechanisms (such as DNA methylation) play a central role since they mediate gene-by-environment interactions. Methylation of the growth-promoting gene *epidermal growth factor receptor* (*egfr*) was indeed shown to control worker size in a highly polymorphic ant. However, it remains unknown if *egfr* methylation could also regulate worker size in monomorphic species. By combining experimental pharmacology and molecular biology, we show that worker size is associated with *egfr* methylation in two monomorphic ants. Furthermore, we functionally demonstrate that EGFR signalling affects worker size. These results indicate that worker size regulation by *egfr* methylation has been mechanistically conserved in ants but remains unexploited in monomorphic species.

## Introduction

Phenotypic plasticity occurs when a single genotype encodes multiple, diverse phenotypes^1^. Common in all living organisms, it is often highly adaptive. By altering their phenotypes in response to external cues, organisms can react to environmental changes, boosting survival and reproduction. Some of the most remarkable forms of phenotypic plasticity are found in social Hymenoptera (bees, wasps, and ants) where the same genotype can lead to morphologically, physiologically, and behaviourally distinct female castes: large, fertile queens that specialise in reproduction, and smaller, usually sterile workers that ensure colony maintenance^2^. Whether a female larva develops into a reproductive queen or a non-reproductive worker typically results from environmental (e.g., food quantity or quality) or social (e.g., queen and brood presence/absence) cues, which cause the baseline genome to express itself along different developmental lines^3–5^.

Unlike bees and wasps, numerous ant species exhibit a high degree of worker polymorphism, i.e. a colony’s workers can vary in size and/or morphology^6,7^. Worker size variation exists along a spectrum, ranging from monomorphism, where there are slight isometric differences, to dimorphism, where there are multiple, distinct worker subcastes that display marked, non-proportional differences in body features. Worker polymorphism has appeared repeatedly in Formicidae, suggesting that size-dependent division of labour in workers promotes colony fitness^8–10^.

Decades of research have been devoted to understanding the evolution and maintenance of worker polymorphism in ants. Yet, its genetic and developmental origins are only beginning to be deciphered. At the molecular level, body size is regulated by evolutionarily conserved growth-regulating pathways, such as the insulin/insulin-like growth factor signalling (IIS)^11^, target of rapamycin (TOR)^12^, and epidermal growth factor receptor (EGFR) signalling^13^. The pathways are triggered by dietary cues, causing cell-signalling cascades that promote cell growth, proliferation, and differentiation^14^. In insects, the result is the production of the growth-regulating hormones ecdysone^15^ and juvenile hormone (JH)^11,13,16^. However, it remains unknown how these pathways have become fine-tuned to generate worker size variation from the same genotype. Recently, it has been hypothesised that epigenetic mechanisms, such as DNA methylation, are at play because they mediate gene-by-environment interactions, translating environmental signals into long-lasting changes in gene expression without modifying the DNA itself^17–19^. Numerous studies have investigated whether DNA methylation influences queen-worker caste determination. In ants^20,21^ and the honey bee^22,23^, queens and workers have different DNA methylation patterns that are associated with differential gene expression and/or alternative splicing. In the honey bee, silencing the expression of the *DNA-methyltransferase 3* gene, which is responsible for de novo DNA methylation, causes worker-destined larvae to become more queen-like^24^. However, multiple studies have failed to detect any caste-specific methylation signatures in ants^25^, wasps^26^, and honey bees^27–29^.

To our knowledge, a single study has explored how DNA methylation could affect worker size variation in social Hymenoptera. In the polymorphic ant *Camponotus floridanus*, which has highly variable sized workers, larval DNA methylation has been shown to regulate adult worker size. The growth-promoting gene *epidermal growth factor receptor* (*egfr*) is differentially methylated and expressed in larvae destined to become large workers (i.e., majors) versus small workers (i.e., minors)^29^. Furthermore, adult worker size can be changed by pharmacologically altering larval DNA methylation: when larvae undergo genome-wide hypomethylation, they grow into larger workers. In contrast, hypermethylated larvae develop into smaller workers. In this species, the methylation status of *egfr* generates most of the size variation within the worker caste^30^*.*

**Figure 1:**
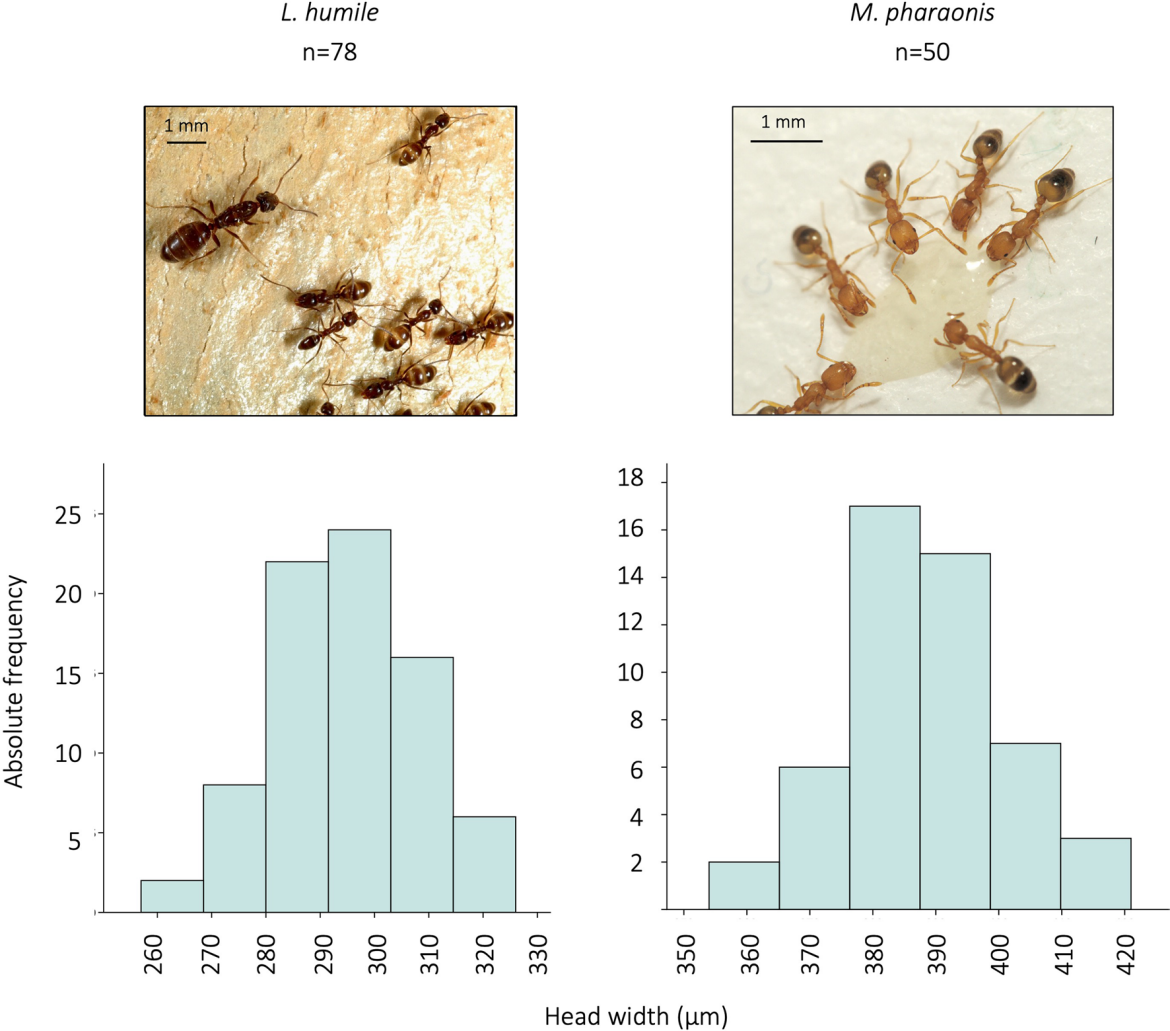
Worker size distributions for *Linepithema humile* and *Monomorium pharaonis*. The plots show the absolute frequencies of workers with different head widths. Both species exhibit a unimodal worker size distribution (i.e., workers are monomorphic). Images: *L. humile*: ©Phil Lester, used with permission; *M. pharaonis*: Зeмлepoйкин, CC BY-SA 4.0.

Here, we show that pharmacological alterations of *egfr* methylation is associated with worker size in two monomorphic ants belonging to distinct subfamilies, the Argentine ant *Linepithema humile* (subfamily *Dolichoderinae*) and the pharaoh ant *Monomorium pharaonis* (subfamily *Myrmicinae*) (Fig. 1), suggesting that worker size regulation by *egfr* methylation has been mechanistically conserved in the Formicidae.

## Results

We first confirmed that the worker caste in *Linepithema humile* and *Monomorium pharaonis* is monomorphic. Worker size distribution was based on measurements of adult workers sampled from the field or laboratory stock colonies, respectively (Fig. 1).

**Figure 2:**
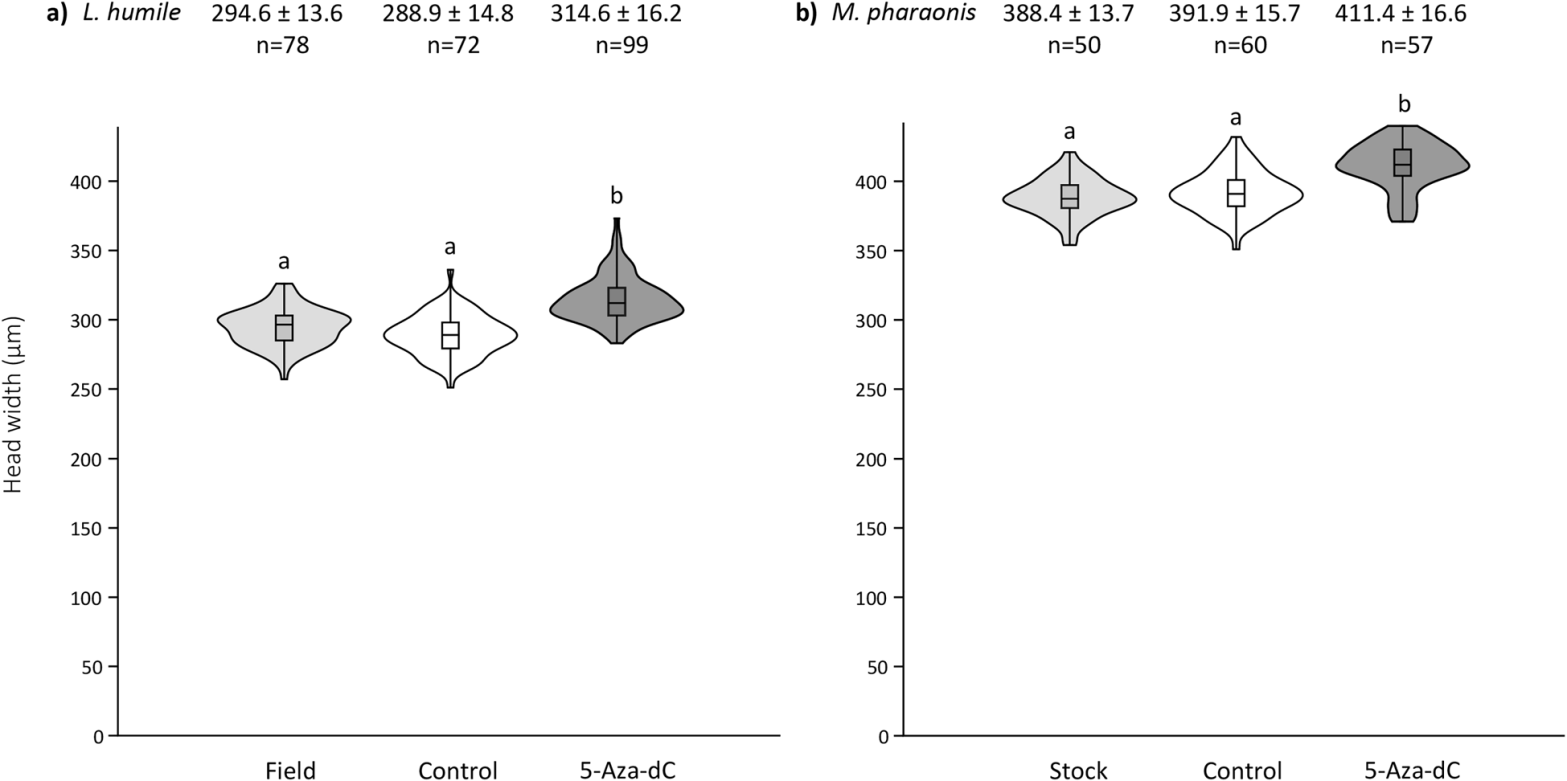
Effect of 5-Aza-dC treatment on worker head width. Violin plots of (**a**) *Linepithema humile* and (**b**) *Monomorium pharaonis* callow head width. Workers were either sampled on the field (*L. humile*) or form laboratory colonies (*M. pharaonis*), or grew from experimental larvae given a control solution (1 M sucrose) or 5-Aza-dC during the first larval instar. Mean head width ± SD and sample size are indicated above each plot. The box’s midline indicates the median; the box’s lower and upper edges are the first and third quartiles, respectively. The whiskers reflect the extreme values. The shapes around the boxes are the kernel density distributions of the data. Differences in the letters above the violins indicate statistically significant differences in worker head width (Kruskal–Wallis test: *p* < 0.0001; Dunn’s post-hoc test with Bonferroni correction: *p* < 0.0001).

To address the role that DNA methylation plays in the regulation of worker size in monomorphic ants, we fed larvae with the pharmacological hypomethylating agent 5-Aza-2’-deoxycytidine (5-Aza-dC)^31^. In both *L. humile* and *M. pharaonis*, adult worker size was significantly greater for larvae given 5-Aza-dC versus the control solution (Fig. 2; Electronic Supplementary Table S2). Notably, 5-Aza-dC also altered the shape of the size distribution in both species; distribution appears skewed to large sizes as compared to the controls in *L. humile*, but to small worker size in *M. pharaonis* (Fig. 2; Electronic Supplementary Tables S2).

**Figure 3:**
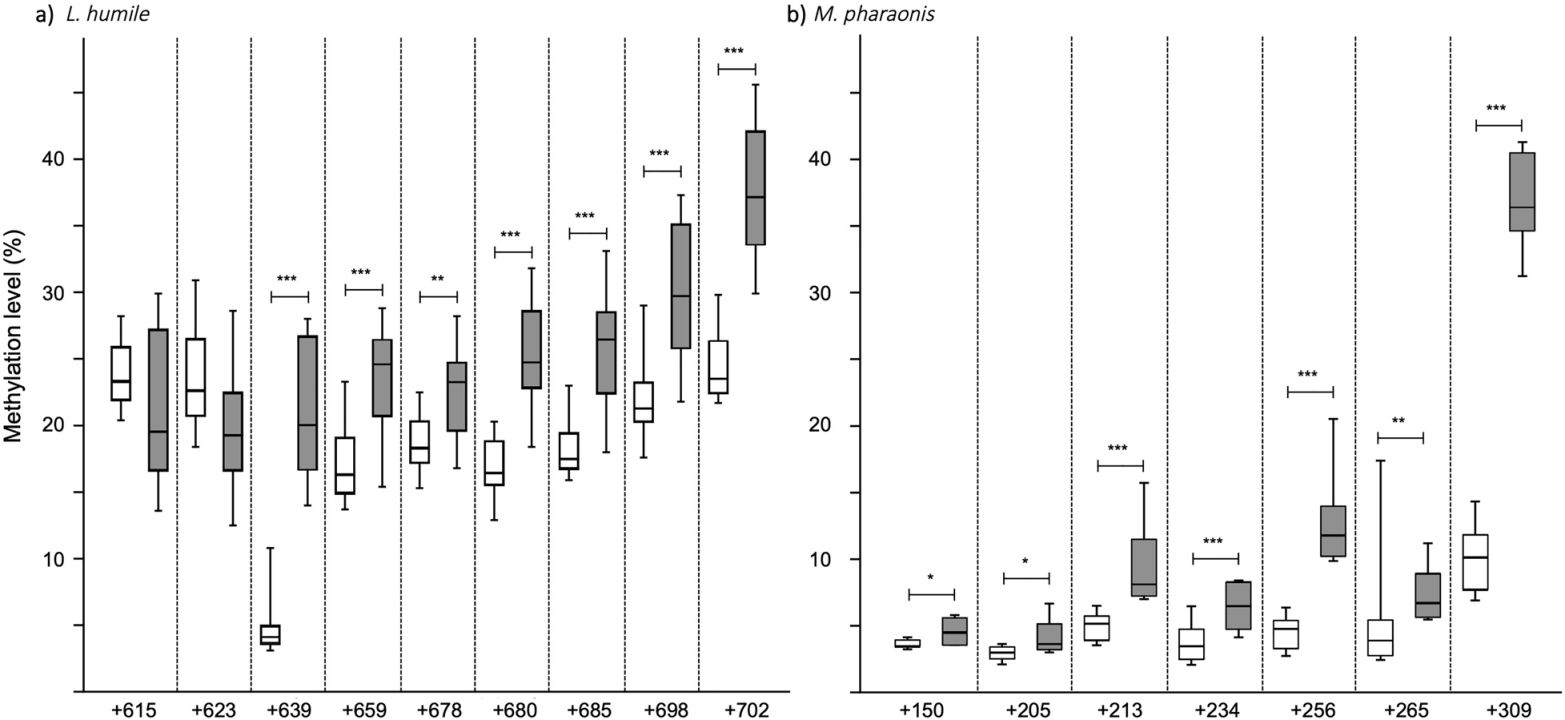
Effect of 5-Aza-dC treatment on *egfr* methylation. Box plots of *egfr* CpG methylation levels for (**a**) *Linepithema humile* and (**b**) *Monomorium pharaonis* prepupae fed a control solution (1 M sucrose; in white) or 5-Aza-dC (in gray) when they were first-instar larvae. The CpGs are numbered from the beginning of the last exon for each species. Each box represents data for at least 8 samples; each sample was a pool of 10 prepupae. The box’s midline indicates the median; the box’s lower and upper edges are the first and third quartiles, respectively. The whiskers reflect the extreme values. Significant differences between the control and 5-Aza-dC-treated groups are indicated: *p < 0.05, **p < 0.01, ***p < 0.005 (Student’s *t* test or Mann–Whitney *U* test).

Since EGFR signalling plays a central role in controlling growth^32^ and *egfr* methylation regulates adult worker size in a polymorphic ant^30^, we then tested if treatment with 5-Aza-dC affected *egfr* methylation. In social Hymenoptera, DNA methylation mostly occurs in cytosine-phosphate-guanine dinucleotides (CpGs) of exons^33,34^. Because ants only grow during the larval development, we measured the methylation levels of several exonic CpGs of *egfr* at the prepupal stage, which marks the end of the larval development. For both species, *egfr* methylation was indeed affected by 5-Aza-dC; each CpG analysed was hypermethylated in the 5-Aza-dC versus the control prepupae (Fig. 3; Electronic Supplementary Table S4).

**Figure 4:**
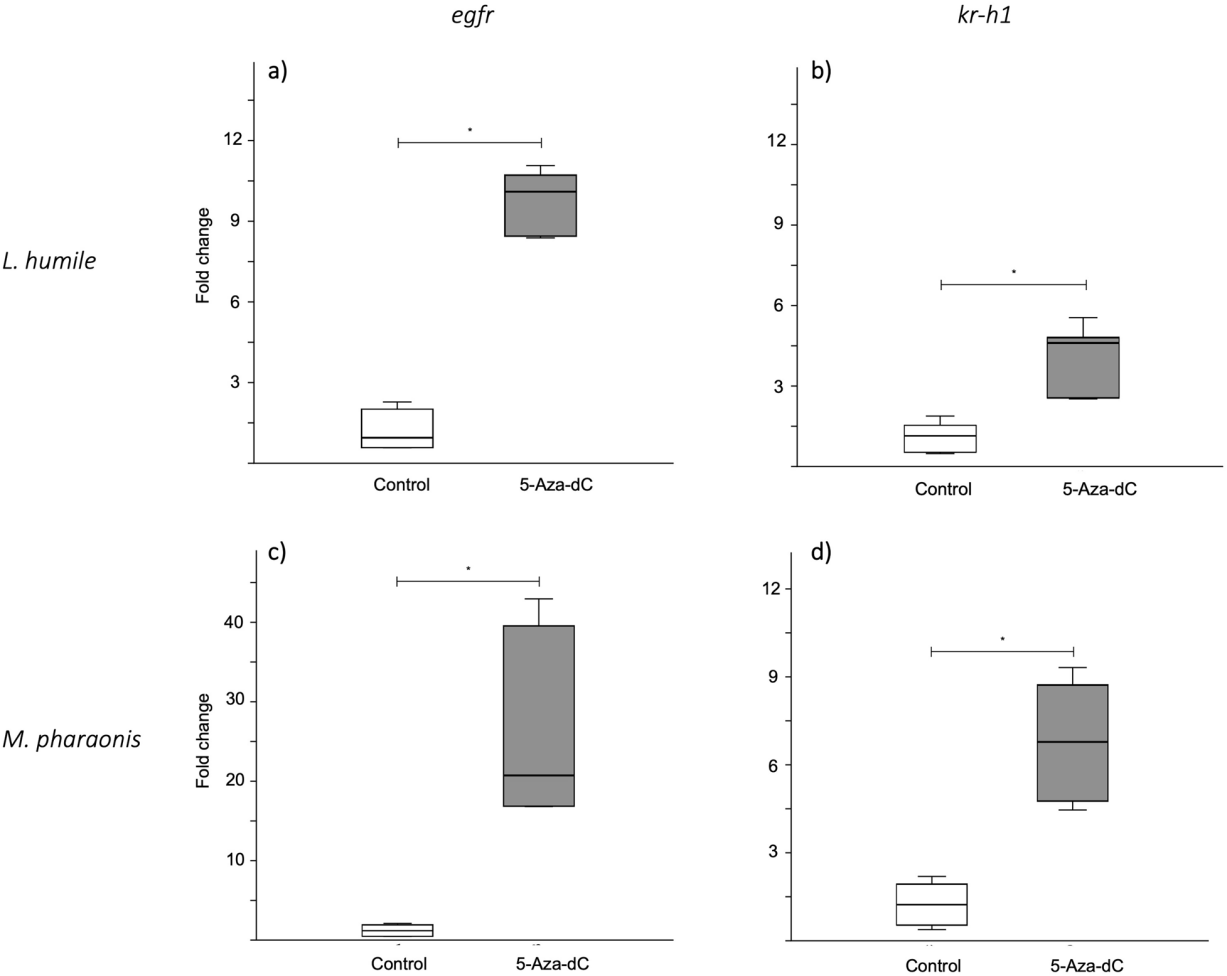
Effect of 5-Aza-dC treatment on *egfr* and *kr-h1* expression. Box plots of relative fold changes in (**a,c**) *egfr* and (**b,d**) *kr-h1* expression for (**a,b**) *Linepithema humile* and (**c,d**) *Monomorium pharaonis* prepupae fed a control solution (1 M sucrose; in white) or 5-Aza-dC (in gray) when they were first-instar larvae. Each box represents data for 6 samples; each sample was a pool of 10 prepupae. The box’s midline indicates the median; the box’s lower and upper edges are the first and third quartiles, respectively. The whiskers reflect the extreme values. Significant differences between the control and 5-Aza-dC-treated groups are indicated: *p < 0.01 (Mann–Whitney *U* test).

Comparison of the expression levels of *egfr* showed that it was significantly overexpressed in 5-Aza-dC-treated prepupae (mean fold change ± SD: *L. humile*—control = 1.18 ± 0.78, 5-Aza-dC = 9.88 ± 1.18, *p* < 0.01; *M. pharaonis*—control = 1.23 ± 0.78, 5-Aza-dC = 26.07 ± 11.59, *p* < 0.01; Fig. 4; Electronic Supplementary Table S5), showing that the pharmacological treatment affected *egfr* expression.

EGFR signalling is known to regulate body size by affecting JH titers^11^, which in turn regulate the expression of the anti-metamorphic transcription factor *krüppel-homolog 1 (kr-h1*) in insects^35,36^. Therefore, we compared the expression of the JH-responsive gene *kr-h1* between control versus 5-Aza-dC-treated prepupae. As for *egfr*, we found that 5-Aza-dC-treated prepupae overexpressed *kr-h1* as compared to controls (*L. humile*: control = 1.13 ± 0.55, 5-Aza-dC = 4.12 ± 1.27, *p* < 0.01; *M. pharaonis*: control = 1.24 ± 0.81, 5-Aza-dC = 6.79 ± 2.01, *p* < 0.01; Fig. 4; Electronic Supplementary Table S5), which means JH titres were higher following 5-Aza-dC feeding.

**Figure 5:**
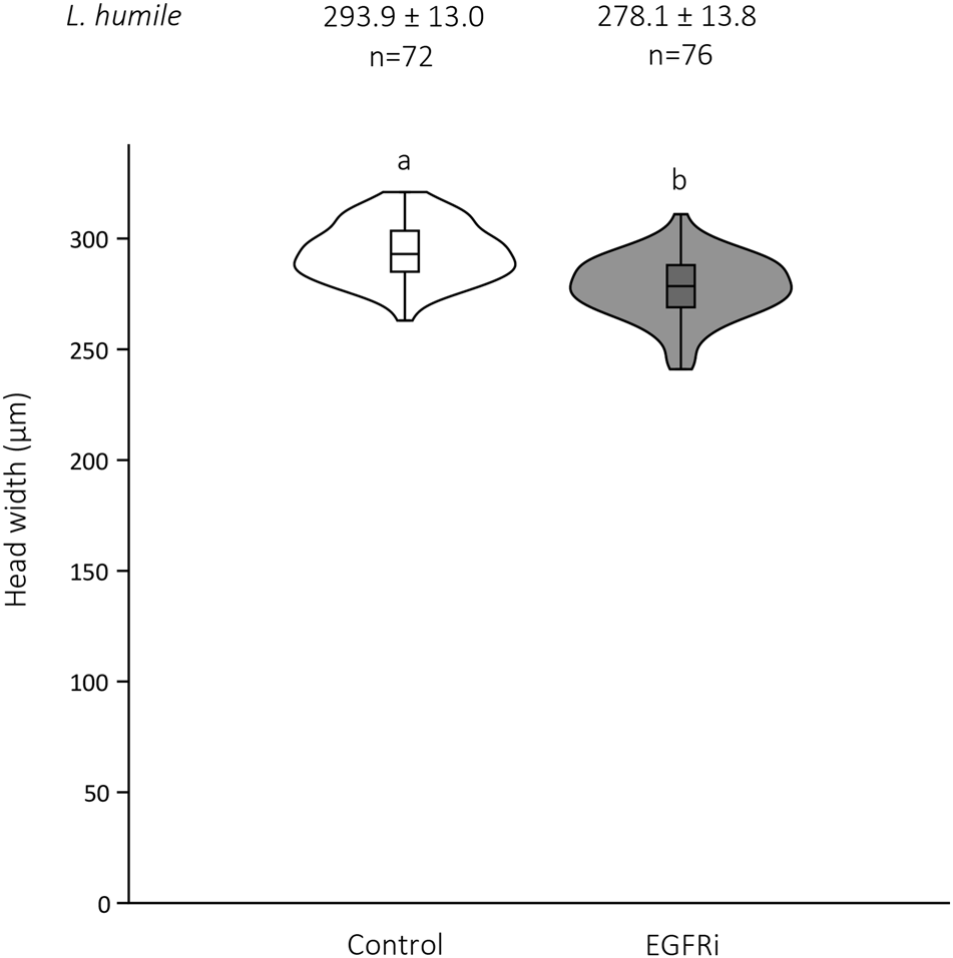
Effect of EGFRi treatment on worker head width. Violin plots of the head widths of *Linepithema humile* callows. Workers came out of two groups: larvae fed a control solution (1 M sucrose [diluted in DMSO]) versus larvae fed EGFRi (diluted in DMSO) during the first-instar stage. Mean head width ± SD and sample size are indicated above each plot. The box’s midline indicates the median; the box’s lower and upper edges are the first and third quartiles, respectively. The whiskers reflect the extreme values. The shapes around the boxes are the kernel density distributions of the data. Differences in the letters above the violins indicate statistically significant differences in worker head width (Student’s *t* test: *p* < 0.0001).

To confirm the specific influence of EGFR signalling on worker size, we fed larvae with PD153035, a specific inhibitor of the EGFR protein (EGFRi) (see methods). As expected, treated larvae developed into significantly smaller adult workers than did the control larvae (*L. humile* only—Student’s *t* test: *p* < 0.0001; Fig. 5; Electronic Supplementary Table S6).

Thus, feeding larvae with the hypomethylating agent 5-Aza-dC increased levels of *egfr* methylation, *egfr* expression, and *kr-h1* expression, which are known to be involved in size regulation^30,32,35,36^. Consistently, the treatment increased the adult size of workers in both species.

## Discussion

These results show that worker size is associated with *egfr* methylation in two monomorphic ant species; they complement the previously established relationship between *egfr* methylation and worker size in a species with polymorphic workers^30^. We found that worker size could be pharmacologically increased in both *L. humile and M. pharaonis* by feeding larvae a hypomethylating agent, 5-Aza-dC. Although the size increase was relatively limited in magnitude, this result highlights that worker size is more plastic than observed under natural conditions. Prior research has noted the same trend in other ant species^37–41^. For instance, the ant genus *Pheidole* is dimorphic: workers are either small (minors) or large (majors). When late-instar *Pheidole* larvae were treated with JH, adult worker size dramatically increased and “super majors” emerged, a novel subcaste barely seen in nature^39^. It is intriguing that worker size plasticity does not appear to be fully exploited under normal ecological conditions. Indeed, polymorphic workers are thought to promote colony fitness by enhancing the division of labour^6–8^. Even in monomorphic ants, workers that vary slightly in size were found to display distinct behaviours^40–43^, suggesting that even limited size variation may be functionally adaptive. It could be that worker size plasticity is constrained under natural conditions because there is a trade-off between worker quantity and quality, where smaller, lower-quality workers are cheaper to create than larger, higher-quality workers^9^. This explanation fits with Argentine ant and pharaoh ant ecology. Both species are highly invasive, and their competitive dominance is largely mediated by worker abundance^44,45^. In the case of a trade-off, investing in larger workers could put colony fitness at risk.

When *L. humile* and *M. pharaonis* larvae were given 5-Aza-dC*, egfr* was hypermethylated at several CpGs in prepupae. It seems counterintuitive that a hypomethylating agent would lead to *egfr* hypermethylation. However, the effect of 5-Aza-dC on DNA methylation has been shown to be sequence-specific^46^. Previous research in social Hymenoptera has found that 5-Aza-dC can indeed cause local hypermethylation^30,47–49^. For instance, in *C. floridanus*, worker larvae fed 5-Aza-dC exhibited both genome-wide hypomethylation and local-level *egfr* hypermethylation^30^. A hypermethylating effect of 5-Aza-dC was also reported in plants^50^. This inverse relationship is thought to be mediated by crosstalk with other epigenetic mechanisms, such as histone post-translational modifications^51^ or non-coding RNAs^52^.

In both species, 5-Aza-dC feeding induced *egfr* overexpression. This result is consistent with the well-established positive relationship between *egfr* expression and growth^53,54^. The fact that worker size decreased in response to the EGFRi treatment confirms that EGFR signalling is directly involved in growth regulation in *L. humile*.

We found that the 5-Aza-dC treatment resulted in the overexpression of *kr-h1* in *L. humile* and *M. pharaonis*, which also concurs with the growth-promoting effects of JH in ants. When JH is produced during specific periods of hormone sensitivity, larval moults replace metamorphic moults, lengthening larval development and ultimately boosting adult body size^55^. Here, we indeed observed that the 5-Aza-dC treatment slightly increased the duration of larval development in both species (see Electronic Supplementary Table S3). To achieve more pronounced developmental shifts, it may be necessary to use higher 5-Aza-dC concentrations or differently timed treatments. Furthermore, the tandem overexpression of *egfr* and *kr-h1* supports the idea that EGFR signalling and JH production are positively correlated. However, because 5-Aza-dC likely affects the methylation of other genes as well, we cannot conclude that *egfr* overexpression was solely responsible for *kr-h1* overexpression. For instance, the insulin/insulin-like growth factor signalling (IIS) pathway promotes JH production in social Hymenoptera^5^, and *kr-h1* overexpression could result from IIS upregulation by 5-Aza-dC. That said, cross-activation occurs between EGFR and receptors of other growth-regulating pathways, which means that EGFR integrates signals prompted by multiple stimuli^56,57^. Therefore, the *kr-h1* overexpression that we observed most likely resulted from crosstalk among several upregulated growth-promoting pathways, including the EGFR signalling system.

Taken together, our results indicate that *egfr* methylation and expression is consistently associated with worker body size in two monomorphic ants belonging to different subfamilies. Two important points must be stressed. First, we examined the effect of *egfr* methylation on worker size, not on caste determination. Indeed, queen-worker caste determination involves many phenotypic changes beyond body size. Thus, our work does not speak to caste determination. Second, although our study indicates that the association between *egfr* methylation and body size is conserved in ants, we did not demonstrate a direct causal relationship between *egfr* methylation and worker size. Notably, other genes may be participating as well. Further complementary work combining genome-wide DNA methylation analyses with emerging methylome editing technologies^58^ should make it possible to unequivocally establish causal links between DNA methylation and the diverse phenotypic traits underlying the ecological success of social Hymenoptera.

## Methods

### Sampling and rearing

*Linepithema humile* were collected in the field in Giens (southern France); *Monomorium pharaonis* were obtained from laboratory colonies reared at Copenhagen University (Department of Biology, Ecology, and Evolution)*.* For both species, we set up large stock colonies that contained several dozens of queens, thousands of workers, and brood at all developmental stages. The colonies were maintained under laboratory conditions (*L. humile*: temperature = 25 ± 1 °C and relative humidity =  ± 30%; *M. pharaonis*: temperature = 28 ± 1 °C and relative humidity = 50%; both: 12:12 light/dark cycle). Colonies were given sugar water ad libitum and received fresh cockroaches every other day.

### Morphometric analyses

To quantify worker size variation, we measured worker head width (eyes excluded), an accurate proxy of worker size in ants^59–61^. All measurements were performed to the nearest 0.001 mm using a MZ6 stereomicroscope (Leica Microsystems, Wetzlar, Germany).

### Pharmacological modification of DNA methylation

Larvae were individually fed 0.5 μl of 10 mM 5-Aza-dC (diluted in sucrose; Sigma-Aldrich, A3656), or 0.5 μl of 1 M sucrose (control solution). Then, 30 treatment or 30 control larvae were transferred into individual experimental nests containing 300 workers but no queens or brood. The experimental nests were checked every day, and emerging workers (*i.e.,* callows) were removed and stored in 99% EtOH for later morphometric analysis. Since workers are completely sterile in both species, we were certain that the callows had developed from the experimental larvae.

The results revealed that the 5-Aza-dC treatment only significantly affected adult worker size when applied to the first-instar larvae (Supplementary Fig. S1; Electronic Supplementary Table S1). We therefore focused on first-instar larvae when repeating the 5-Aza-dC experiment for both species. Half of the larvae were left to develop into adults for the morphometric analyses. The other half were collected at the prepupal stage, flash frozen, and stored at −80 °C for subsequent methylation and gene expression analyses. The use of the prepupal stage for molecular analyses stems from the finding that 5-Aza-dC altered the developmental duration in both species studies. Therefore, by comparing control and 5-Aza-dC larvae at a specific developmental stage (prepupae), we ensured that the signal detected resulted from treatments (control *vs* 5-Aza-dC feeding), rather than from differences in developmental rates.

### DNA methylation

The methylation of *egfr* was measured using direct bisulfite sequencing (dBS)^62–64^. We pooled ten individuals from each group to obtain enough biological material. Each pool was treated as a biological replicate.

We extracted genomic DNA using an SDS/proteinase K in-house protocol, which was followed by phenol–chloroform/chloroform washes and ethanol/sodium acetate precipitation. Genomic DNA fragmentation was assessed using agarose gel electrophoresis. Quantity and absorbance ratios were measured using a Qubit 2.0 fluorometer (Thermo Fisher Scientific) and a NanoDrop 1000 spectrophotometer (Thermo Fisher Scientific), respectively. Genomic DNA was treated with sodium bisulfite to convert unmethylated cytosines to uracils without affecting the methylated cytosines^65^. Bisulphite conversion was performed on 200-ng samples of genomic DNA using a Methylamp DNA Modification Kit (Epigentek, P-1001) in accordance with the manufacturer’s instructions.

We designed primers using MethPrimer v. 2.0 software^66^ with a view to amplifying a CpG-rich region in the last exon of *egfr* after bisulfite conversion*.* The sequence for *egfr* was obtained from the Hymenoptera Genome Database^67^. The PCR reactions consisted of 10 μl of 2× Multiplex Mastermix (Qiagen, 206143), 2 μl of 10 μM forward and reverse primers, 5 μl of ddH_2_O, and 1 μl of bisulphite-treated genomic DNA. Cycling conditions were as follows: 15 min at 95 °C; 35 cycles of 30 s at 94 °C, 90 s at 54 °C, and 90 s at 72 °C; and 10 min at 72 °C. Because bisulfite conversion reduces DNA complexity and therefore decreases PCR specificity, we extracted the target PCR products directly from agarose gels after carrying out overnight migration using a QIAquick Gel Extraction Kit (Qiagen, 28706).

We directly sequenced the gel-extracted PCR products using a 3730 DNA Analyser (Thermo Fisher Scientific) and a BigDye Terminator v. 3.1 Cycle Sequencing Kit (Thermo Fischer Scientific, 4337457). The sequencing reactions consisted of 2 μl of PCR products, 6 μl of ddH_2_O, 1 μl of BigDye, 2.1 μl of BigDye 5× buffer, and 0.2 μl of 10 μM forward primer. Each biological replicate was sequenced using technical triplicates. The methylation levels of each CpG were measured using CodonCode Aligner v. 9.0.1 software (CodonCode Corporation).

### Gene expression

We measured *egfr* expression using reverse transcription quantitative real-time PCR (RT-qPCR). Total RNA was extracted using TRI Reagent (Thermo Fischer Scientific). RNA fragmentation, quantity, and absorbance ratios were assessed as described above. The relative expression of *egfr* and *kr-h1* were quantified utilising the ΔΔCt method^68^ and *rpl32* for normalisation^69,70^. The sequences were obtained from the Hymenoptera Genome Database^65^, and the primers were designed using NCBI primer BLAST software^71^.

### Inhibition of the EGFR signalling pathway

First-instar larvae of *L. humile* were fed PD153035 (EMD Millipore, 234491), a compound that specifically inhibits EGFR tyrosine kinase activity, which is needed to activate the EGFR pathway, allowing downstream signalling, cell growth, and cell proliferation^72^. EGFRi has already been used successfully in another ant species^30^. The treatment larvae were given 0.5 μl of 10 μM EGFRi (diluted in 1 M sucrose and DMSO). The control larvae were given 1 M sucrose (diluted in DMSO). Each group’s larvae were then transferred into individual experimental nests. Callows were collected, and their body size was measured within a day of emergence.

### Statistical analyses

We tested for data normality and heteroscedasticity using the Shapiro–Wilk test and Levene’s test, respectively. An appropriate statistical approach was then employed (parametric vs. non-parametric). All the statistical analyses were performed using Past4 software v. 4.10^73^.

## Supplementary Information

Supplementary Tables.

Supplementary Figure 1.

## Data Availability

All data generated or analysed during this study are included in this published article [and its supplementary information files].
