# Supplementary material for: DNA methylation and expression of the egfr gene are associated with worker size in monomorphic ants
Source: Sci Rep. 2022 Dec 8;12:21228. doi: 10.1038/s41598-022-25675-4 (PMC9732050; doi:10.1038/s41598-022-25675-4)
Supplement: Supplementary file 2 — Supplementary Figure 1. [file 41598_2022_25675_MOESM2_ESM.docx]

**Supplementary Fig. 1. Effect of 5-Aza-dC treatment during different larval stages (instars 1, 2, or 3) on worker head width in *Linepithema humile*.** Box plots of callow head width for larvae fed a control solution (1M sucrose; in white) or 5-Aza-dC (in gray) during the first-, second-, or third-instar stage. Mean head width ± SD and sample size are indicated above each plot. The box’s midline indicates the median; the box’s lower and upper edges are the first and third quartiles, respectively. The whiskers reflect the extreme values. Differences in the letters above the boxes indicate statistically significant differences in worker head width (Kruskal-Wallis test: *p* < 0.0005; Dunn’s post-hoc test with Bonferroni correction: *p* < 0.0005).
